# Low Abundance Taxa Show Diverse Microbial Symbiotic Interactions With the Freshwater Sponge, *Radiospongilla crateriformis* , Pre and Post Gemmulation

**DOI:** 10.1111/1758-2229.70331

**Published:** 2026-04-06

**Authors:** Taylor A. Strope, Cole G. Easson, Cara L. Fiore

**Affiliations:** ^1^ Department of Biology Appalachian State University Boone North Carolina USA; ^2^ Department of Biology Middle Tennessee State University Murfreesboro Tennessee USA

## Abstract

Freshwater sponges, most of which have a dormant stage with gemmules, are well poised for microbiome focused experiments. Here, we leveraged field collections of freshwater sponges pre‐gemmule (Pre) and post‐gemmule (Post) formation to compare the microbial symbiont metatranscriptome at the two developmental stages. There were no broad changes to the microbiome in composition between the two stages; however, there were significant differences in the abundance of several bacterial taxa and functional genes between Pre and Post sponge samples. For example, many *Polynucleobacter* spp. increased from Pre to Post samples, but no putative symbiosis factors were associated with *Polynucleobacter* and these may be loosely associated with the sponges. In contrast, we hypothesise that *Flavobacterium* spp. are facultative symbionts of freshwater sponges that begin to leave when the sponge tissue degrades, or they may decrease their metabolic activity. Functions attributed to *Flavobacterium* spp. such as type IX secretion system (T9SS) component and ankyrin repeat domains, all decreased in the Post samples and suggests that this group can interact with the sponge host or be free‐living. These results provide a foundation for future hypothesis testing and experimental work with the microbiomes of freshwater sponges.

## Graphical abstract

The microbiome profile of freshwater sponges did not broadly change in taxonomy or functional profile based on metatranscriptome analysis prior to and after formation of sponge dormant bodies (gemmules). Results highlight a subset of taxa that likely associate with the sponge host in different ways from facultative to long‐term symbionts.

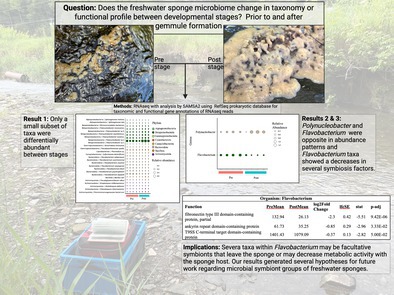

## 1 Introduction

Sponges are in Phylum Porifera, sessile, filter feeding animals that first appeared in the fossil record approximately 600 million years ago (Love et al. 2009). While there are many diverse forms and lineages of marine Porifera, only one major lineage is present in freshwater worldwide: the order Spongillida (Class Demospongiae, subclass Heteroscleromorpha) (Manconi and Pronzato 2008; de Voogd et al. 2024). Many freshwater sponges can tolerate extreme chemical and physical conditions (e.g., desiccation, cold temperatures), largely due to an asexual life cycle that includes a dormant stage called gemmules (Fell 1990; Gugel 2001; Manconi and Pronzato 2007, 2016b).

Gemmules are both a form of asexual reproduction and a cryptobiosis stage for sponges (Manconi and Pronzato 2016b). The formation of gemmules is typically driven by winter conditions or drought and environmental factors of temperature, pH, conductivity, likely influence the timing of gemmule formation and hatching of new sponge individuals from the gemmules (Calheira et al. 2019, 2020). Freshwater sponges also can host unicellular algae and/or other microbial symbionts that may have a role in host ecology (e.g., Gaikwad et al. 2016; Sugden et al. 2022). However, the characterisation of the microbiome of freshwater sponges has lagged behind that of marine sponges. For example, there is limited knowledge about the acquisition of microbial symbionts in freshwater sponges (Sugden et al. 2022; Paix et al. 2024; de Fernando et al. 2025). Additionally, freshwater sponges are generally considered to be low microbial abundance (LMA, microbial densities ~10^5^–10^6^ cells g^−1^ sponge wet weight) sponges (e.g., Gernert et al. 2005; Costa et al. 2013), in contrast to high microbial abundance (HMA, 10^8^–10^10^ g^−1^ sponge wet weight; Hentschel et al. 2003), but to our knowledge, no cell densities of freshwater sponges have been published. Recent work in this system, however, has used widely distributed freshwater sponge species of *Spongilla lacustris* or *Ephydatia muelleri* in experimental studies of vertical transmission of prokaryotic and/or algal symbionts (Hall et al. 2021; Paix et al. 2024).

Like marine sponges, freshwater sponges harbour a microbiome that is distinct from the surrounding water (Manconi and Pronzato 2007; Sugden et al. 2022; Keleher et al. 2025). Additionally, like marine sponges, there is high consistency in the microbiome across geography for a given species, with minor but still detectable differences driven by local environmental conditions (Sugden et al. 2022; de Fernando et al. 2025). However, while there does not seem to be high similarity between the water or abiotic surface communities and corresponding freshwater sponge microbiomes (Gaikwad et al. 2016; Laport et al. 2019; Sugden et al. 2022; Paix et al. 2024; de Fernando et al. 2025), similarity of these microbial communities may vary over the life cycle of the sponge (Paix et al. 2024; Keleher et al. 2025). Specifically, the microbiome profile of *Radiospongilla crateriformis* from the New River in North Carolina, USA, showed high overlap with that of the river water when the sponge first emerged for the season and then diverged from the water column community over time, suggesting some influence of the host and/or microbes on the microbial symbiont community composition (Keleher et al. 2025).

Microbial symbionts of metazoans can have a significant role in the development of host structure, immunity, diet and ecological niche (Reviewed by Nyholm and McFall‐Ngai 2004; Manconi and Pronzato 2008; Douglas 2011; Hentschel et al. 2012; Brestoff and Artis 2013). For example, symbiotic bacteria influenced tissue proliferation in lab‐reared hydra, which were unable to proliferate by budding in individuals sterilised of their microbiome and that effect was reversed when inoculated with a hydra‐derived microbiome (Rahat and Dimentman 1982). In freshwater, as with marine sponges, most microbial symbionts are considered commensals, but experimental work on defining diverse symbiont‐derived benefits to the host (i.e., beyond photosymbionts, reviewed in Freeman et al. 2021) and/or role in host development (e.g., Turon et al. 2024) is still in its infancy. In freshwater sponges, aside from work on algal symbionts (e.g., Frost and Williamson 1980; Geraghty et al. 2021; Hall et al. 2021; Hustus et al. 2023), work towards understanding the prokaryotic community, which appears to be primarily Bacteria with few Archaea (Sugden et al. 2022; Hustus et al. 2023; Paix et al. 2024; Keleher et al. 2025) is limited. One metagenome analysis has been published using the model sponge for many evolutionary developmental studies (e.g., Elliott and Leys 2007; Mitchell and Nichols 2019), *Ephydatia muelleri* (Sugden et al. 2022). Typical symbiont traits in the *E. muelleri* microbiome such as eukaryotic‐like domains and an abundance of defence genes were observed, while the genetic repertoire for degrading many sponge‐derived compounds was inconsistent (Sugden et al. 2022). The latter observation was surprising and may underscore our limited understanding of this symbiont system. The diversity of freshwater sponge microbiomes varies across species (Gaikwad et al. 2016; Laport et al. 2019; de Fernando et al. 2025), with *Ephydatia muelleri* and several species in western North Carolina having relatively low symbiont diversity (Sugden et al. 2022; Keleher et al. 2025). Thus, many freshwater sponges, with their relatively simple microbial symbiont community and potential as an experimental system for microbiome work given that gemmules can be hatched in the laboratory, may provide unique insight into host‐symbiont interactions. We use the term symbiont here to refer to sponge‐associated microbes regardless of their beneficial, negative, or neutral interaction with the host (Oulhen et al. 2016).

Previous work in western North Carolina has shown that freshwater sponges may select for a subset of microbes present in the water as the sponges grows (Keleher et al. 2025) and therefore may have specific taxa that are important to certain developmental stages. As a step towards understanding the potential role of microbial symbionts in sponge development, we set out to first investigate how the composition of freshwater sponge microbial communities may change as the sponge undergoes developmental changes involved in asexual reproduction (i.e., formation of gemmules). We leveraged samples that were collected prior to and after the formation of gemmules in an RNAseq experiment to generate metatranscriptomes that were used to compare the taxonomic and functional profile of sponge symbionts prior to and after gemmule formation (Pre and Post samples, respectively).

## 2 Experimental Procedures

The overall experimental design is a correlative study using field‐collected samples that spanned 2 years of collections at one site. The original design included experimental manipulation of gemmules in the laboratory (winter 2018), but due to inconsistent hatching rates, this was removed from the design, and we focused on a correlative field study in 2019. In the fall of 2019, most sponge samples collected did not contain gemmules and we lacked enough replicates from post‐gemmule formation to conduct the RNAseq experiment; we therefore included sponge samples collected from both 2018 and 2019 (Table 1). The goal of the experiment was to assess transcriptional changes in the microbial symbiont community between the two distinct developmental time points of prior to formation of gemmules (‘Pre’ samples) and post formation of gemmules (‘Post’ samples). Previous microbiome profiling work suggested that there were taxonomic shifts in the microbial community in sponges collected from the same site over time from early vegetative growth to mid/late vegetative growth to gemmule formation (Keleher et al. 2025). Therefore, with this work we aimed to further investigate potential changes in microbial activity between the vegetative growth (pre gemmule stage) and asexual reproductive (post gemmule stage) stages of freshwater sponges.

**TABLE 1:** Metadata for samples used in the present study, including developmental stage as pre‐ or post‐gemmule formation, year and month in which samples were collected, and individual sample ID number and collection site.

Stage	Year	Sample ID	Month	Collection site
Pre	2018	NR16	July	New river
Pre	2018	NR17	July	New river
Pre	2019	NR50	July	New river
Pre	2019	NR55	August	New river
Post	2018	NR24	August	New river
Post	2018	NR25	August	New river
Post	2019	NR57	September	New river
Post	2019	NR58	September	New river

### 2.1 Field Collections

Freshwater sponge samples were collected over 3 months (July, August, September) in 2018 and 2019 from New River State Park in Ashe County, North Carolina (36.4615° N, 81.3387° W) under permit 2021_0548. From personal observations, sponges in western North Carolina are not found over the winter and instead grow during summer months of June, July, and early August, then form gemmules in late August or September, although there is some variation by sponge species and location (Keleher et al. 2025). Therefore, sampling was conducted during the time of sponge growth and gemmule formation from July to September. Sponges were preserved in RNAlater (Invitrogen, USA) for metatranscriptome analysis. In 2018, four individuals were collected between July and August. In 2019, four individuals were collected during July, August, and September (Table 1). In the field, samples were identified as either containing gemmules or not (i.e., Pre or Post gemmule formation) and this was confirmed by light microscopy (dissecting microscope) with a portion of the sample when it was processed for RNA extraction. Each sample for the metatranscriptome analysis was collected using a sterile razor blade to scrape a portion of the sponge off the rock, or substrate, into a sterile microcentrifuge tube filled with 2 mL of RNAlater. Tubes were placed on ice until return to the laboratory at Appalachian State University (~1 h). Samples were stored at −80°C, until further analysis.

Sponge species identification was accomplished by spicule analysis as in Keleher et al. (2025). Spicule preparations were conducted by dissolving a small piece of sponge in bleach overnight in a microfuge tube. The spicules were rinsed with distilled water, then mounted on a slide to view with a compound microscope. The taxonomic keys of Reiswig et al. (2010) and Manconi and Pronzato (2016a) were used to identify the sponges. Megascleres and gemmuloscleres (when present) were observed with brightfield microscopy for taxonomic identification.

Environmental data of pH, temperature, and conductivity were recorded at the New River site using an Oakton ORPTestr 50 handheld metre (Cole‐Parmer, Vernon Hills, IL, USA) in July, August, and September of 2019 at the time of each collection. Additionally, in 2019, 1 mL of water was preserved with 50 μL of 10% paraformaldehyde to a final concentration of 0.5% for flow cytometry. Preserved samples were stored on ice until return to the laboratory and moved to −80°C for storage until shipping on dry ice to the Center for Aquatic Cytometry at Bigelow Ocean Sciences (Boothbay, ME, USA). Due to logistical constraints, no environmental data were collected in 2018. At Bigelow, a ZE5 Cell Analyser flow cytometer (Bio‐Rad, Hercules, CA, USA) was used to measure optical properties of single cells from each sample and quantify requested populations. To ensure accurate calibration of the flow cytometer, ZE5 QC beads (Bio‐Rad, Hercules, CA, USA) were run daily.

Picophytoplankton (less than 3 μm) and nanophytoplankton (3–20 μm) were analysed using a slight modification of the method described in Lomas et al. (2010). Immediately after thawing at room temperature, 300–400 μL of sample was prescreened through 70 μm mesh and run at a flow rate of 1 μL s^−1^. Particles were excited with a 488 nm blue laser and data acquisition was triggered on red fluorescence. Signals were recorded from detectors with bandpass filters for forward scatter (FSC), right angle light scatter (SSC) and fluorescence emission in red (692/80 nm) indicative of chlorophyll *a*, and orange (593/52 nm) for phycoerythrin. Data files were analysed from logarithmic dot plots based on fluorescence and characteristic light scattering properties (Durand and Olson 1996) using FlowJo 10.6 software (Becton Dickinson & Company, San Jose, CA, USA). Total pico‐ and nanophytoplankton populations were identified based upon cell size and red fluorescence. Phycoerythrin containing cell populations were determined by orange fluorescence. Based upon these gating criteria, the number of cells in each identified population was enumerated and converted to cell abundances using the processed sample volume and adjusted for dilution by preservative.

For total bacteria analysis, samples were thawed, diluted 1:10 with Tris EDTA (TE) Buffer pH 8.0 and stained using a 10× working stock of SYBR Green I Nucleic Acid Stain (Thermofisher Scientific, USA) at room temperature in the dark for 15 min using the protocol of Marie et al. (2005). At a flow rate of 0.5 μL s^−1^, 180 μL of the diluted sample was run. Particles were excited with a 488 nm blue laser and data acquisition was triggered on green fluorescence. Signals were recorded from detectors with bandpass filters for forward scatter (FSC), right angle light scatter (SSC) and fluorescence emission in green (525/35 nm). Data files were analysed from two logarithmic scatter plots based on fluorescence and characteristic light scattering properties. Total bacteria counts were identified based on size and presence of green fluorescence and counts were converted to cell abundances using the volume of sample processed including adjustments for preservation, dilution and staining. Differences in bacterial and phytoplankton concentrations were tested with a Welch's *t*‐test for each month, followed by a test correction using the methods of (Benjamini and Hochberg 1995). Analysis of variance (ANOVA) was used to assess the effect of month on phytoplankton and bacterial concentrations, while sample size was small (*n* = 3), with appropriate testing for assumptions of the statistical tests performed.

### 2.2 RNA Extraction and Sequencing

Extraction of total RNA from each of the freshwater sponge samples was performed using a TRIzol Reagent (Sigma Aldrich, USA) per the manufacturer's protocol for simultaneous extraction of RNA and DNA. Extractions were conducted in November of 2019, soon after the fall collections were complete. The extracted RNA was then used in DNase I Treatment (Promega, WI, USA) to remove genomic DNA and Monarch RNA Cleanup Kit to remove protein and other contaminants (New England Biolabs, MA, USA). DNA extraction was performed using the TRIzol Reagent protocol from the same samples as for RNA extraction and archived for later use. The concentration and quality of RNA was analysed via NanoDrop 2000. The selection of RNA products for sequencing was based on quality ratios of 260/280 with values upwards of 2.0 or greater, except samples NR16 and NR17 which had values of 1.60 and 1.35 respectively. The extracted RNA was processed by GeneWiz (NJ, USA) to analyse quality and concentration by a bioanalyzer, remove eukaryotic ribosomal RNA (rRNA), which generally comprises the majority of RNA in a total RNA pool (Ribo‐Zero rRNA removal kit, Illumina, CA, USA), construct the paired‐end cDNA library, and sequence on one lane of an Illumina HiSeq 2000 (150 nt read length). All extracted DNA products were low in concentration (average 69 ng/μL) and were therefore selected based on the absorbance ratio of 260/280 value of typically greater than 1.40.

### 2.3 Metatranscriptome Analysis

RNA sequence analysis was performed using the SAMSA2 pipeline (Simple Analysis of Metatranscriptomes through Sequence Annotation), version 2.0 (Westreich et al. 2018) on a high‐performance cluster computer (Intel Xeon CPU E5‐2697 v2 2.70GHz with 256 GB RAM). The SAMSA2 programme is focused on analysing RNAseq reads and no assembly of the reads was conducted in this study. The pipeline consists of a set of python scripts that follow three main processing steps: preprocessing of reads to remove and trim low quality reads, annotation of the reads, and aggregation of reads for downstream processing (Westreich et al. 2018). More details on SAMSA2 processing can be found in the Supporting Information (S1). The resulting reads contained both sponge and microbial RNA reads. Reads were mapped to the freshwater sponge genome of *Ephydatia muelleri* (Kenny et al. 2020) to determine the proportion of sponge and non‐sponge reads, but we focused our work on the microbial symbiont (i.e., bacterial) metatranscriptome. For metatranscriptome analysis, the reads were mapped to the RefSeq bacterial genomic database (downloaded from NCBI on March 20, 2020) for annotation within the SAMSA2 pipeline. RefSeq annotation provided both taxonomic and functional annotation for the reads.

All statistical analyses were performed in R (R Core Team 2023) using RStudio version 3.6.3. The PCA ordinations and PERMANOVA were performed in R using the packages ‘stats’ with function ‘prcomp’ and the package ‘vegan’ (Oksanen et al. 2025) with the ‘adonis2’ function for the PCA and PERMANOVA, respectively. Specific taxa that were identified as differentially abundant between the two stages were then used in organism‐specific DESeq2 analysis. The taxonomic and functional profile plots were made with the ggplot2 package in R (Wickham 2016). SAMSA2 scripts (Westreich et al. 2018), were used to calculate Shannon and Simpson diversity indices and principal coordinates analysis (PCA) analysis. Also, within SAMSA2, DESeq2 (Love et al. 2014) was used to calculate differential features (i.e., taxa or functions) between Pre and Post sponge samples.

## 3 Results

**FIGURE 1:**
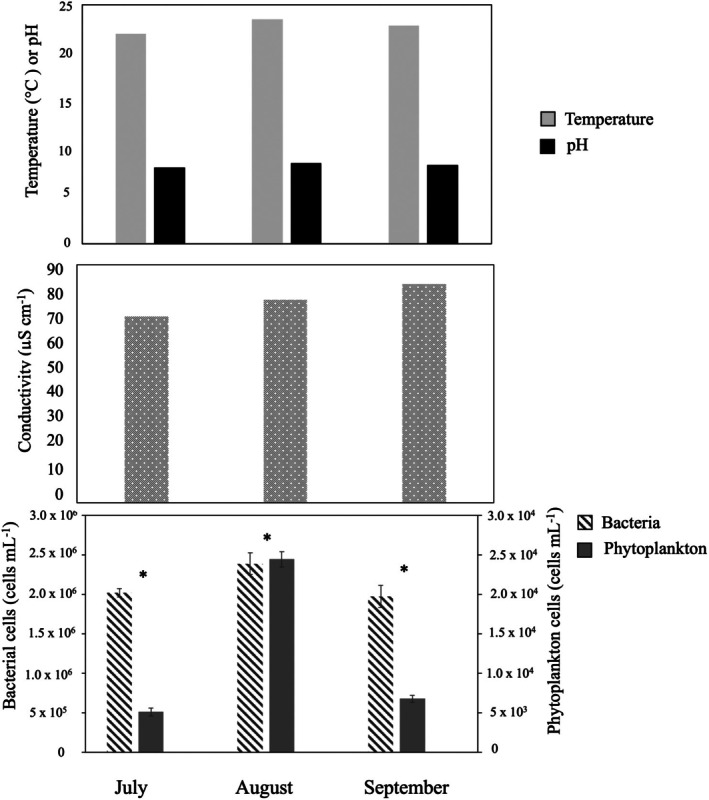
Water environmental data collected before and during the sampling period in 2019 in the New River. One measurement of temperature, pH, and conductivity was recorded for each month of sampling, while three replicate samples were used for bacterial and phytoplankton cell counts. Significant differences between phytoplankton and bacterial cell counts are noted with an asterisk (*t*‐test, adj. *p* < 0.05). There was also a significant effect of month on bacterial cell density (ANOVA, *F*_2,6_ = 11.2, *p* = 0.009) and on phytoplankton cell density (ANOVA, *F*_2,6_ = 773, *p* < 0.001). Note that phytoplankton cell density is on a second *Y*‐axis.

Morphological and spicule preparations of the collected sponges indicate a putative identification of the sponges as *Radiospongilla crateriformis* (Potts 1882) for all samples, with characteristic oxeas with subtle spines for megascleres and gemmuloscleres of birotules with curved spines on each rotule and several spines at the terminal ends of the rotule shaft as in Manconi and Pronzato (2016a) (Figure S1). Environmental data from 2019 indicated little change in temperature and pH during the sampling months (July–September), while conductivity increased steadily from July to September (Figure 1). Bacterial cell concentrations were always significantly higher than phytoplankton concentrations (Figure 1, *t*‐test, adj. *p* < 0.05). There was a significant effect of month on bacterial cell density (Figure 1, ANOVA, *F*_2,6_ = 11.2, *p* = 0.009) and phytoplankton cell density (ANOVA, *F*_2,6_ = 773, *p* < 0.001).

**FIGURE 2:**
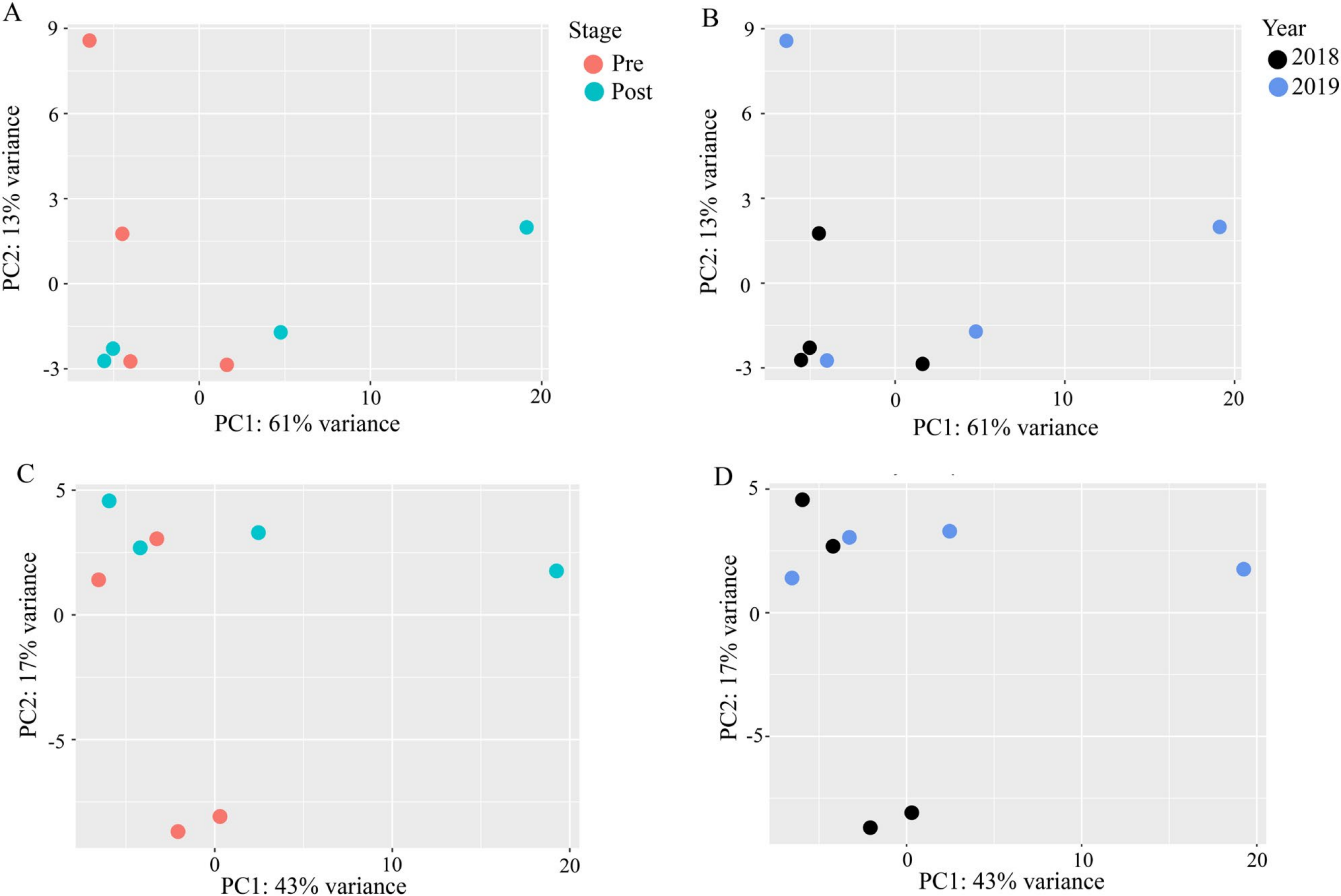
Ordination by principal components analysis (PCA) of Pre and Post Samples by RefSeq functional (A, B) and RefSeq organismal (i.e., taxonomic; C, D) annotation. Ordinations are coloured by sponge developmental stage (A, C) where Pre and Post labels correspond to pre‐ and post‐gemmule formation of the sponges, and by year collected (B, D).

For the metatranscriptome, total reads generated per samples ranged from 37,446,129 to 54,321,279 (Table S1). Between raw reads and final read counts, the percentage lost was < 31% for all samples with an average loss of 25% after the merging of paired reads and removal of low‐quality reads (Table S1). The proportion of reads that mapped to the RefSeq prokaryotic database was smaller (11%–16% of reads) than the amount that mapped to the freshwater sponge genome of *Ephydatia muelleri* (28%–46% of reads, Table S2). Shannon and Simpson diversity metrics were similar between Pre and Post sponge samples (Table S3). For organismal results the Shannon diversity was ~6 for all samples, while the Simpson diversity was ~0.98 for all samples. For functional results, Shannon diversity was ~4.6 for all samples, and the Simpson diversity was ~0.88 for all samples. Ordination by PCA did not indicate separation by stage (Pre and Post) or by year of collection for functional profile (i.e., RefSeq functional gene annotations; Figure 2A,B) or for organismal profile (i.e., RefSeq taxonomic annotations; Figure 2C,D). The lack of separation of sponge samples by stage and by year is supported by the results of PERMANOVA between each of the two groups (*F*_1,7_ = 1.2, *p* = 1.3 (stage), *F*_1,7_ = 1.2, *p* = 0.18 (year) for function; *F*_1,7_ = 1.1, *p* = 0.32 (stage), *F*_1,7_ = 0.87, *p* = 0.58 (year) for organism).

**FIGURE 3:**
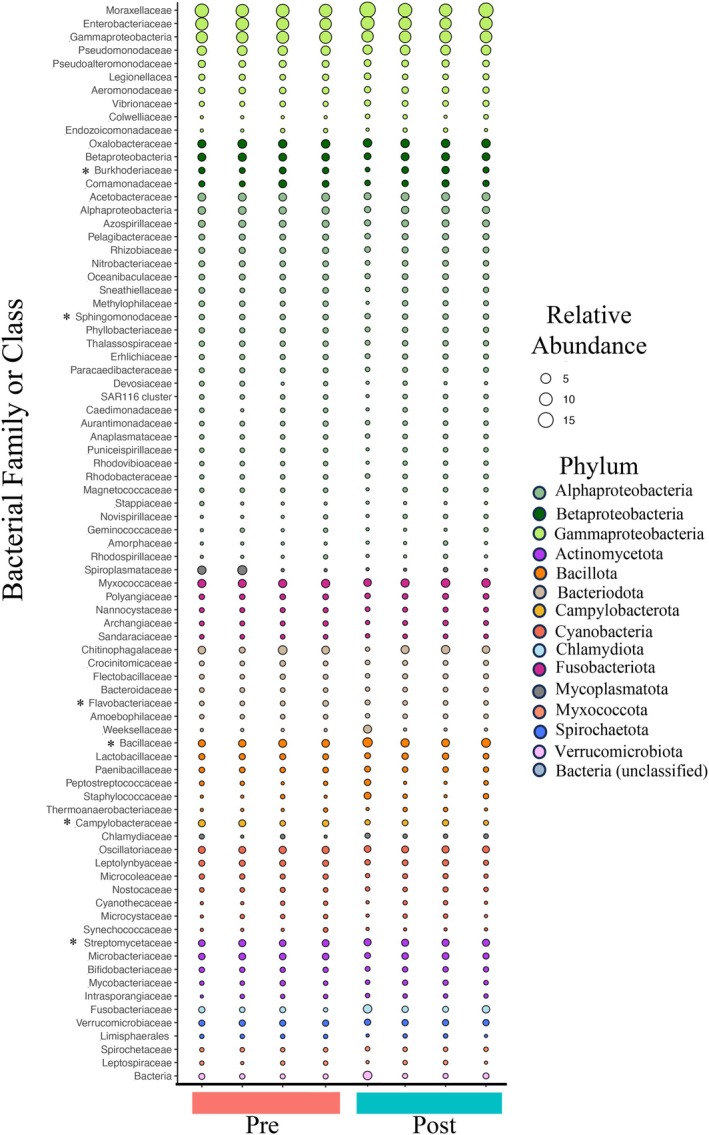
Relative abundance of taxonomic families across sponge samples for taxa with 10% or greater abundance in each sample derived from the metatranscriptome. Relative abundance values represent percentages. Asterisks mark taxa that contained differentially abundant taxa (not all groups are included that were differentially abundant as some were low abundance taxa). Taxonomy is derived from taxonomic annotation of RNA reads using the RefSeq prokaryotic database. Pre and Post labels correspond to sponges pre‐ and post‐gemmule formation in sponges.

**FIGURE 4:**
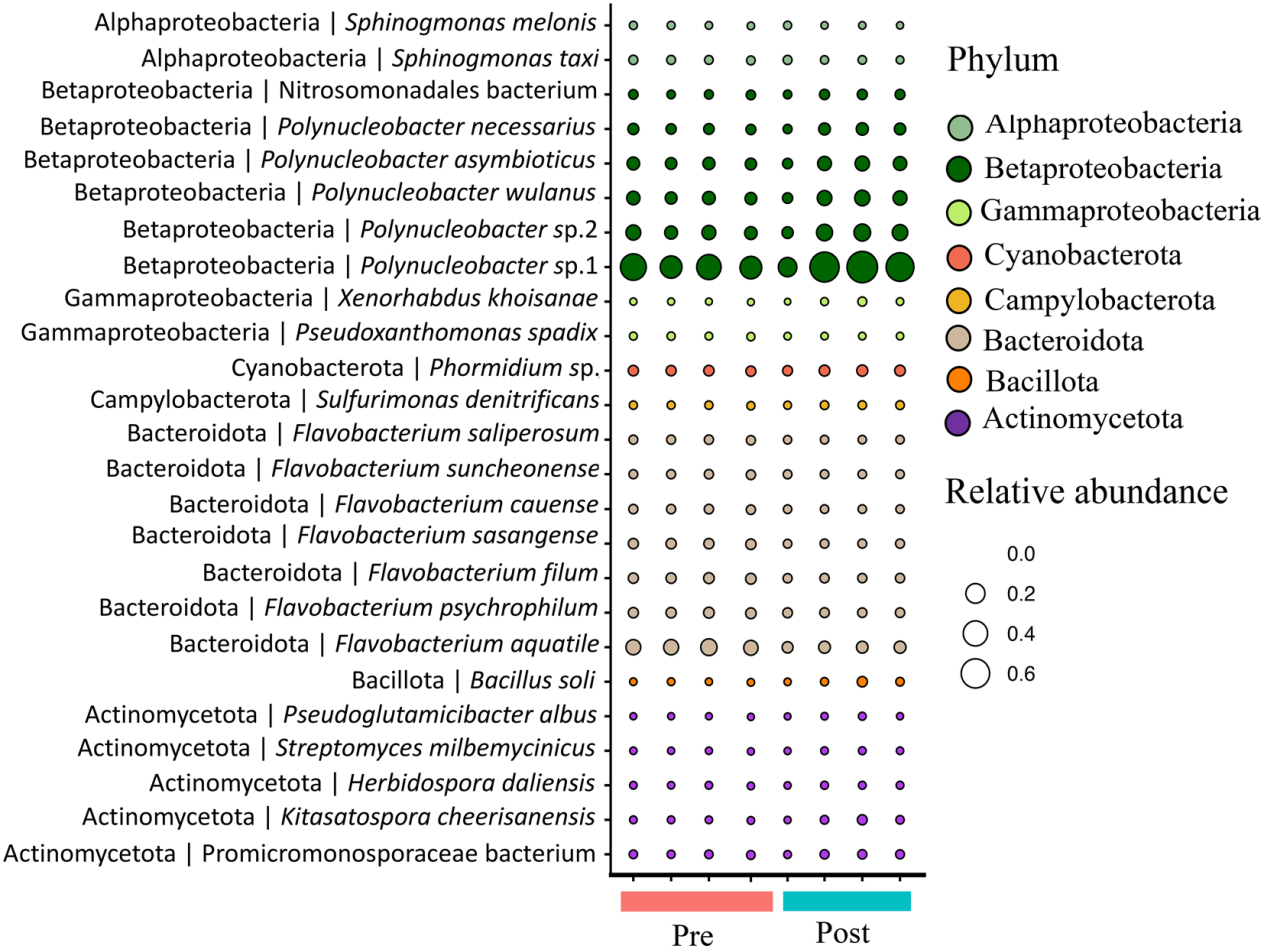
Relative abundance of differentially abundant taxa between sponge stages (Pre and Post). Relative abundance values represent percentages. Taxonomy is derived from taxonomic annotation of RNA reads using the RefSeq prokaryotic database. Pre and Post labels correspond to sponges pre‐ and post‐gemmule formation in sponges. The results of the differential abundance analysis are provided in Figure S3 and Table S5.

**FIGURE 5:**
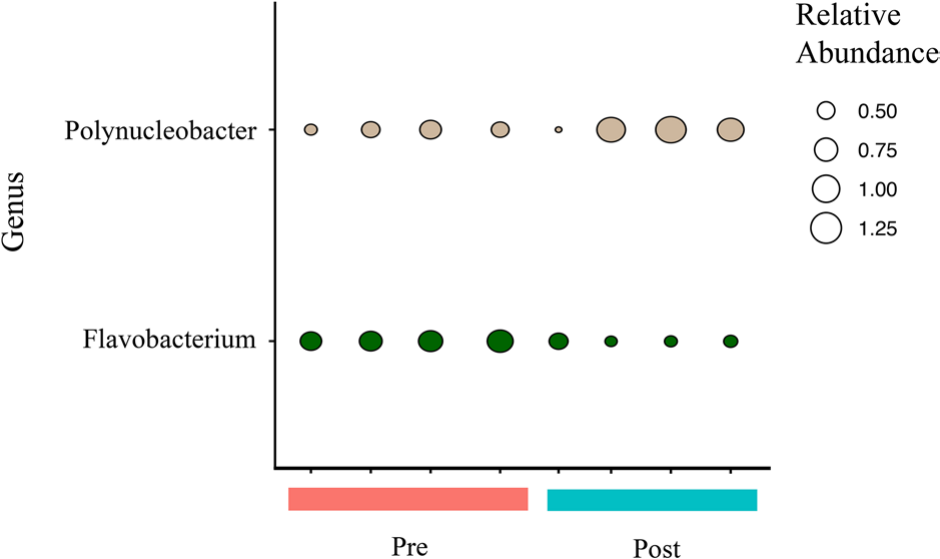
Relative abundance of RNAseq‐derived reads with the taxonomic annotation of the genus *Flavobacterium* or *Polynucleobacter* in each sample. Samples for pre‐ (Pre) and post‐ (Post) gemmule formation are indicated. Relative abundance values represent percentages.

The most prevalent taxa across all sponge samples based on the metatranscriptome analysis included taxa mainly within the Gamma‐, Beta‐, and Alphaproteobacteria (Phylum Pseudomonodota), as well as taxa within Bacteroidota, Bacillota, and Myxococcota (Figure 3, Table S4). Most taxa when grouped at the family level were similar in relative abundance across samples, including groups such as Alpha‐, Beta‐ (contains *Polynucleobacter*), and Gammaproteobacteria, Flavobacteriaceae (contains *Flavobacterium*), Synechococcaceae, and Verrucomicrobiaceae that would be expected in freshwater sponges (Figure 3). However, some taxa such as Spiroplasmataceae, Chitinophagalaceae (includes *Sediminibacterium* shown in Figure S2), Bacillaceae, and Camplyobacteraceae visibly differed in relative abundance across samples but not necessarily consistently between stages, at least at the family level (Figure 3). While there were not consistent differences between Pre and Post samples at the family level, DESeq2 analysis revealed differential abundance between stage (Pre and Post) for the following taxa at the genus level: Actinobacteria including taxa within the Family Streptomycetaceae (i.e., *Kitasatospora* and *Streptomyces* genera), *Flavobacterium* within the Bacteroidota phylum, Betaproteobacteria such as *Polynucleobacter* spp., Alphaproteobacteria such as *Sphingomonas* spp., a Campylobacterota, *Sulfurimonas*, and one taxon within Firmicutes (*Bacillus* spp.) (Figures 4, S3; Table S5). While these taxa were significant (Figure S3; Table S5), there were generally small changes in relative abundance across samples (Figure 4). Multiple *Flavobacterium* and *Polynucleobacter* species were significant and in general, *Flavobacterium* spp. decreased while *Polynucleobacter* spp. increased in abundance from the Pre to Post stage of gemmule production (Table S5; Figures 5 and S3).

**FIGURE 6:**
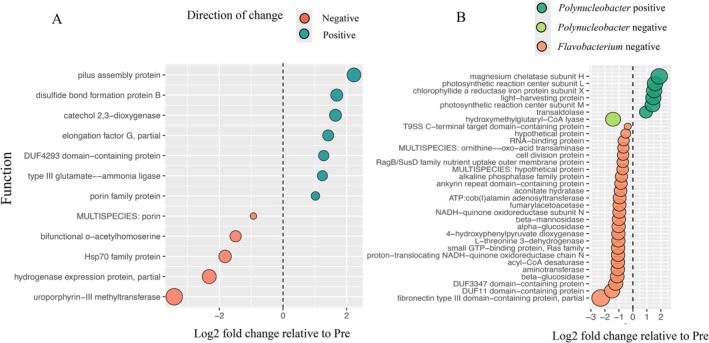
The log2 fold change in abundance for functions that were significant in the differential abundance analysis of RNA reads. Significant functions from all reads (A) and significant functions from reads assigned to the genus *Polynucleobacter* or *Flavobacterium* (B). Pre samples were used as the control, so a positive fold change indicates functions that increased in abundance in Post samples, and a negative fold change indicates functions that decreased in abundance in the Post samples. Function was derived from the functional annotation of RNA reads using the RefSeq prokaryotic database. Results of the differential abundance analysis are provided in Tables S7 and S8.

The largest proportion of RefSeq annotated functions were proteins of unknown function, followed by energy and maintenance metabolism (e.g., electron transport chain enzymes and citric acid cycle enzymes), genetic information processing (e.g., translation machinery, chaperone proteins), cellular processes (e.g., focal adhesion proteins), and signalling, which primarily included transporters (Figure S4; Table S6). Most of the general metabolic processes such as carbohydrate metabolism, amino acid metabolism, and oxidative phosphorylation, as well as translation and transcription processes, and protein folding, were similar in proportion of reads across Pre and Post stages (Figure S4; Table S6). However, 12 functions were found to be differentially abundant between Pre and Post samples, with five functions that decreased and seven that increased (Table S7; Figure 6A). Functions that increased in abundance in Post relative to Pre samples included reads for catechol 2,3‐dioxygenase, involved in aromatic compound degradation, porin proteins, which are channel proteins common in Gram negative bacteria, disulphide bond formation protein B, which catalyses disulphide bond formation in periplasmic proteins (Gram negative bacteria), enzymes in nitrogen metabolism (type III glutamate—ammonia lyase), pilus assembly protein, a domain of unknown function (DUF4293), and an elongation factor involved in proteins translation and ribosome recycling (EF‐G) (Table S7; Figure 6A). In contrast, functions that decreased in abundance in the Post samples included reads involved in vitamin B_12_ bioynthesis (Uroporphyn‐III methyltransferase), amino acid metabolism (bifunctional *o*‐acetylhomoserine/*o*‐acetylserine sulfhydrylase), as well as different porin proteins, the chaperone protein Hsp70, and a hydrogenase protein (Table S7; Figure 6A).

Lastly, from the list of taxa that showed a significant difference in abundance between Pre and Post gemmule samples (Table S5), four genera were selected for more in‐depth functional analysis: *Polynucleobacter*, *Flavobacterium, Sulfurimonas*, and *Sphingomonas*, as they were the most frequently listed (Table S5) or were of interest for their potential role in nutrient cycling. However, within these four organisms, only two genera contained functional categories that were significantly different from pre and post samples: *Polynucleobacter* and *Flavobacterium* (Table S8). There were seven functions in *Polynucleobacter* that were different, six of which increased in abundance from pre to post gemmule formation and were involved in bacteriochlorphyll biosynthesis or a transaldolase, involved in the metabolism of carbohydrates in the pentose phosphate pathway (Table S8; Figure 6B). In contrast, *Flavobacterium* had 25 functions that all significantly decreased in abundance in samples from pre to post gemmule formation. The *Flavobacterium* functional categories included proteins likely involved in maintenance metabolism (e.g., acyl‐coA ligase in fatty acid metabolism, NADH‐quinone oxidoreductase subunit N) as well as several proteins of unknown function and proteins known to be involved in protein–protein interactions a host (i.e., fibronectin type III domain‐containing protein, ankyrin repeat domain‐containing protein, Type IX secretion system (T9SS C‐terminal target domain‐containing protein)) (Table S8; Figure 6B).

## 4 Discussion

Here we leveraged the natural life cycle of freshwater sponges to gain insight into interactions between the sponge and microbial symbionts. Novel findings from this work include a decrease in relative abundance of several taxa, such as *Flavobacterium* spp. in the post‐gemmule samples as well as an increase in functions that may be related to degradation of complex molecules, motility, and/or host–microbe interactions such as catechol 2,3‐dioxygenase, pilus assembly proteins, type IX secretion system (T9SS) component, ankyrin repeat domains, and fibronectin type III domain components.

### 4.1 Broad Environmental and Taxonomic Patterns Associated With Sponge Asexual Development

The stable pattern of temperature and pH does not suggest a strong influence of these factors on developmental changes in the sponge. Additionally, previous work found consistently low inorganic nutrients at the same site used in this study and did not observe a significant change in water temperature in a nearby creek from July to September (Keleher et al. 2025), suggesting that these parameters are relatively stable during the times of this study. In contrast, conductivity increased each month and was highest at the time of sampling in 2019 for the post‐gemmulation sample, and bacterial and phytoplankton concentrations peaked in August and then decreased in September. These data are limited as they were only collected in 2019, however, it is possible that conductivity and/or particulate organic matter may be a cue for sponge gemmulation. Previous work has documented conductivity as a factor influencing the distribution of sponge species for freshwater sponges (e.g., Jewell 1935; Evans and Montagnes 2019), although to our knowledge, the impact of conductivity on the timing of gemmulation is not known. Other environmental factors not quantified here such as light intensity and duration and dissolved organic matter could also have a role in the sponge life cycle (reviewed by Simpson and Fell 1974). Lastly, it is possible that the change in bacterial cell density of the surrounding water could influence the abundance patterns within the sponge in our dataset, particularly for any transient bacteria present in sponge tissue. We discuss our results in the context of the current literature and focus on hypothesis generation from this work.

In the metatranscriptome analysis, Pre and Post microbial communities did not form two distinct groups based on taxonomy (organismal analysis), suggesting no major restructuring occurred between these stages and there is likely a stable community during the sampled stages. In previous microbiome analysis of this sponge species, compositional changes over time were detected based on 16S rRNA gene profiling, but primarily from first emergence of the sponges to vegetative growth (Keleher et al. 2025). In this study however, we did observe specific taxa present at relatively low abundance that increased in one or the other stage (Pre or Post). One bacterial group that was identified as significantly different in abundance between the Pre and Post samples, overlapped with taxa previously identified as enriched in sponges from this same site, based on 16S rRNA gene profiling (i.e., *Flavobacterium* spp., Keleher et al. 2025). Differences in the methodology for amplicon profiling versus the metatranscriptome profiling as well as differences between profiling DNA versus RNA contribute to some expected differences in taxonomy when comparing our metatranscriptome‐based taxonomy to amplicon studies. However, despite these differences, several parallels in taxonomy were observed although the implications of these findings should be interpreted cautiously. Most taxa within the order Flavobacteriales decreased in abundance from Pre to Post samples, while in previous work, this group included taxa only present in sponges based on 16S gene profiling (not in the river water) and were stable in their relative abundance over time (Keleher et al. 2025). The decrease in relative abundance in the present study is difficult to put in context without absolute numbers but suggests that many of these taxa leave the sponge or decrease their metabolic activity. Interestingly, Flavobacteriacaea were prevalent and increased in relative abundance in *Spongilla lacustris* over time from no gemmules to gemmule formation and degradation of the adult sponge in situ (Paix et al. 2024). Furthermore, Flavobacteriaceae were dominant members of the early hatching and juveniles stages of new sponges, particularly, for those gemmules where the epibacterial community on the outside of the gemmules were removed, indicating a potential role as with attachment and development of the sponge (Paix et al. 2024). Our opposite results with a decrease in abundance in the metatranscriptome may suggest a short‐term relationship with *R. crateriformis* as the sponge host for some taxa of *Flavobacterium*. Alternatively, these bacteria may remain within the sponge tissue or gemmules and decrease their metabolic activity (e.g., Dittmer and Brucker 2021). Further work would be needed to differentiate amongst these options. However, taken together these data support that this group is an important component of the freshwater sponge microbiome.

Most other taxa differed between the previous profiling work (Keleher et al. 2025) and the metatranscriptome analysis in the present study. For example, *Sediminibacterium* spp. (Bacteroidota) were observed in the same species of freshwater sponges as in this study (Keleher et al. 2025) and in other sponge species (e.g., Sugden et al. 2022; Paix et al. 2024), but this genus was not highlighted in the current metatranscriptome work. Different taxa within the Alphaproteobacteria (e.g., *Sphingomonas* spp.) and within the Actinomycetota (e.g., *Promicromonosporaceae*) were also observed in this metatranscriptome study compared to previous work with the same species of sponge (Keleher et al. 2025). It may be that *Sediminibacterium* and many other taxa did not change in abundance in the sponges over time as we were focused on taxa that changed from Pre to Post samples. Limited change in abundance was supported by visualising the relative abundance of reads assigned to the *Sediminibacterium* genus. Two groups that were differentially abundant in the present work and were more abundant in Pre samples include the genera *Streptomyces* sp. and *Xenorhabdus* sp., which are taxa within Alphaproteobacteria that were not highlighted in previous work, but are of interest as symbionts in multiple organisms, including marine sponges (Seipke et al. 2012; Dreyer et al. 2018). It is not clear why the abundance would be higher in the Pre samples for these bacterial groups; it may be that these taxa leave the sponge, or they may decrease their metabolic activity when the sponge begins to degrade. While the specific interactions of these taxa (*Flavobacterium*, *Sediminibacterium*, *Streptomyces*, *Xenorhabdus*) with the sponge host may differ (e.g., commensal, facultative, obligate), they may all represent important bacteria within freshwater sponge hosts and are targets for future work.

### 4.2 Symbiotic Traits and Taxa Associated With Sponge Asexual Development

While there are many nuances in symbiosis and ways to define symbiotic relationships, we can consider three main categories of bacterial life histories that could be present in the sponge samples: (1) free‐living, (2) obligate or long‐term facultative symbiont (we cannot distinguish between these two) that may remain with the sponge as it degrades, and (3) short‐term facultative symbiont that disperses from the sponges when it disappears. These groups are similar to the groups defined in previous work documenting changes in the microbiome of *Daphnia magna* from a healthy state to death (Preiswerk et al. 2018). Below we discuss these three broad life history strategies in the context of our results and generate hypotheses about which of these groups, if any, were observed in the current dataset.

First, if we are detecting free‐living bacteria that happened to be included in the sample from water present in the sponge or as food particles, we might expect to see variation in the presence of taxa between the two sampling points (stages) that may be driven by stochastic environmental factors such as runoff from rain events (e.g., Niño‐García et al. 2016; Huttunen et al. 2025). Because bacterioplankton populations decreased over time in the river water, we would expect to see a decrease in relative abundance of many free‐living taxa. Additionally, heterotrophy would likely be the most common metabolism as this tends to be most prevalent in river bacterioplankton (e.g., Biddanda et al. 2001; Shao et al. 2021), but photosynthetic bacteria are present and previous work in this river indicated low and consistent relative abundances of Cyanobacteria in the water from July to September based on amplicon microbiome profiling (Keleher et al. 2025). In comparison to symbiotic bacteria, we hypothesise that there would likely be limited motility and chemotaxis transcripts, as well as transcripts for transposable elements, viral defence, and proteins with eukaryotic‐like domains (Thomas et al. 2010).

In this regard in our experiment, taxa within the genus *Polynucleobacter* had significant changes in transcript abundance that were only associated with photosynthesis, which increased in the Post samples. The increase in photosynthesis genes is unexpected given the decrease in irradiance from summer into fall and the typical presence of the post‐gemmule stage in early fall, but two of the Post samples were collected in August, which may account for some increase between Pre and Post and this pattern may be driven more by environment than interaction with the sponge. Most taxa within *Polynucleobacter* were also observed to increase from Pre to Post stages in our study. *Polynucleobacter* was originally described as endosymbionts that resided in ciliates (*P. necessaries*, Heckmann and Schmidt 1987), but more recently, multiple *Polynucleobacter* strains were identified as abundant and widely distributed free‐living planktonic bacteria in freshwater systems (Hoetzinger et al. 2019; Borton et al. 2025). There is high ecological diversity of *Polynucleobacter* spp. in freshwater habitats (Hahn et al. 2016) and at least one strain has been characterised with the capacity to do anoxygenic photosynthesis (Martinez‐Garcia et al. 2012). No other symbiotic traits (e.g., eukaryotic‐like domains, chemotaxis) were observed in the *Polynucleobacter* analysis and thus far in the literature there is limited support for *Polynucleobacter* as a sponge symbiont (Gernert et al. 2005; Paix et al. 2024; Keleher et al. 2025). However, this does not preclude some of these strains from being symbiotic; they may be at low abundance and/or may not change their activity with changes in the sponge. Indeed, several of the best matches in NCBI were to the symbiotic *P. necessaries* specifically. We also note that one strain of *Polynucleobacter* was identified as enriched in freshwater sponges from this same site in previous work (Keleher et al. 2025) and this genus was observed in juvenile freshwater sponges hatched in the laboratory (Paix et al. 2024) and in freshwater sponges and river water in the Amazon (de Fernando et al. 2025). Specifically, in the Amazonian freshwater sponge *Metania reticulata* , *Polynucleobacter* is hypothesised to break down DOM in a manner that is beneficial to the sponge host (de Fernando et al. 2025). Further monitoring of sponge and water microbial populations may shed more light on the role of this group.

Second, in contrast to free living bacteria, if we detected long‐term facultative or obligate symbiotic bacteria that associated with the sponge during vegetative growth, these may be intentionally transmitted into the gemmules. Such a relationship would be similar to vertical transmission in many marine sponges which can include high percentages (> 30%) of symbionts that are passed to the offspring through gametes (Webster et al. 2010; Schmitt et al. 2012; Reveillaud et al. 2014). Indeed, several groups of taxa, including Flavobacteriacea, were observed to be vertically transmitted through gemmules in *Spongilla lacustris* (Paix et al. 2024). We did not examine the gemmules for microbiome profiles, but we would expect some traits to be present and potentially change in expression with the corresponding developmental and structural changes in the sponge.

To help define what we would look for in long‐term or obligate symbionts in our dataset, we propose the following possible outcomes for gene expression patterns in such symbionts as the sponges degrades (similar to the patterns of microbial abundance outlined by Preiswerk et al. 2018): (1) Their metabolic activity could show no change over time or may increase as sponge begins to disappear and undergoes structural changes. An increase in metabolic activity may be the result of more nutrients released as the sponge degrades (Preiswerk et al. 2018) or because of interaction with and movement within the sponge. (2) Similarly, expression of nutrient transporters might increase in expression as the sponge begins to disappear due to potential release of metabolites as the sponge degrades. (3) Metabolic functions that could provide certain nutrients (e.g., vitamins, amino acids, organosulfur compounds) to the host may show no change or increase in expression as the host produces gemmules and begins to disappear. (4) Presence of transposable elements and viral defence would be expected to be higher in the symbiotic community than in free living microbes based on marine sponge microbiome analyses (Thomas et al. 2010; Horn et al. 2016) but may show little to no change between stages (Pre and Post) for symbiotic microbes. (5) Disappearance of the sponge tissue may also increase expression of motility and/or chemotaxis genes in symbiotic bacteria as these bacteria respond to remodelling and developmental changes in the sponge. (6) Lastly, expression of proteins with eukaryotic‐like domains has been documented as an enriched trait in sponge and other symbiont communities in comparison to free living microbes (Thomas et al. 2010; Horn et al. 2016; Slaby et al. 2017). Expression of such proteins might decrease upon sponge degradation after gemmules are produced if these bacteria leave the sponge (i.e., facultative symbionts) or may increase if this is involved in transmission into gemmules (i.e., obligate, or at least long‐term, symbionts).

Results from our work that may apply to this second framework would be functions highlighted in Table S7, such as: pilus assembly protein, porin family protein, catechol 2,3‐dioxygenase, and type III glutamate‐ammonia ligase. These functions increased in expression in the Post samples. Pili are structures used to adhere to surfaces, like a host, or in bacteria‐bacteria communication and have been documented in marine sponge symbiotic bacteria (Liu et al. 2011). Therefore, an increase in pilus assembly protein could be evidence of increased bacteria‐bacteria interaction or potentially bacteria‐host interaction in the Post samples. The porin family of proteins is a type of transport protein usually located in the outer membranes of Gram‐negative bacteria, which were the dominant type of bacteria in the New River sponges in the present study and previous work (Keleher et al. 2025). The main function of porins is to import specific molecules into the cell, such as certain types of sugars as well as secondary metabolites, any of which would likely be produced within the sponge (Fan et al. 2012; Kamke et al. 2013). In particular, as the sponge degrades, it may release different metabolites and nutrients, and symbiotic bacteria may be well‐suited to take advantage of such compounds (Preiswerk et al. 2018). The increase in transcripts for catechol 2,3‐dioxygenase in the Post samples may be involved in degradation of aromatic compounds released by the sponge and/or other bacterial symbionts. Type III glutamate‐ammonia ligase, also known as glutamine synthetase, is an important enzyme in nitrogen regulation which forms glutamine from ammonia and glutamate. Nitrogen regulation through the protein PsII, which regulates glutamine synthetase, has been documented as enriched in marine sponge symbiont communities in comparison to free‐living communities (Thomas et al. 2010; Moitinho‐Silva et al. 2017). While we cannot say conclusively here if the pattern we have described is related to symbiont lifestyle, we hypothesise that the observed increase in transcript abundance of these functional traits in the Post samples reflects characteristics of a group of bacteria that have adapted to their host environment and life cycle (e.g., Liu et al. 2011; Preiswerk et al. 2018). We do not have taxonomy for these functional traits and further work here is needed, particularly as these bacteria would be candidates to be transferred into gemmules if there is selective pressure to maintain these lineages with the sponge life cycle.

Third, if we are detecting short‐term facultative symbiotic bacteria that associate with the sponge during vegetative growth and then transition to free‐living after the sponge disappears, we might expect that these bacteria would either leave the sponge to become pelagic again or remain until they are released as the host disappears (Preiswerk et al. 2018). In this case we would expect similar traits to the long‐term symbionts described above, but with some potential differences in response to gemmule formation and degradation of the sponge: (1) Heterotrophs may increase in metabolic activity as the sponge disappears, while autotrophs such as ammonia and sulphur oxidising microbes (e.g., Liu et al. 2012; Moitinho‐Silva et al. 2017; Slaby et al. 2017) may decrease if reliant on host metabolism or increase as sponge tissue degrades, depending on which nutrients are released. (2) The expression of motility and/or chemotaxis genes might increase in expression upon sponge degradation if these bacteria leave the sponge. (3) Expression of proteins with eukaryotic‐like domains, nutrient importers, presence of transposable elements and viral defence, and metabolism that could provide certain nutrients (e.g., vitamins, amino acids, organosulfur compounds) to the host, might decrease in expression as the sponge begins to disappear.

Results from our work that may fit with this third category of symbionts include the functions observed to significantly decrease in expression in the genus *Flavobacterium*. These include: T9SS C‐terminal target domain, fibronectin type III domain‐containing protein, and ankyrin repeat domain. T9SS C‐terminal target domain is a type IX secretion system only found in some species of the *Bacteroidetes* phylum and has one of two roles: a means of gliding movement for commensal or environmental bacteria or involved in virulence secretion by pathogens (Lasica et al. 2017). Interestingly, the non‐pathogenic bacteria with T9SS are known to degrade cellulose or chitin (Lasica et al. 2017), while other secretion systems enriched in the marine sponge symbiont *Entotheonella* included T2SS associated with degradation of collagen (Liu et al. 2016). The ability to degrade collagen was hypothesised to be critical for *Entotheonella* to create space in the sponge mesohyl (Liu et al. 2016). This is the first record of T9SS in putative sponge symbionts and may be involved in nutrient acquisition and/or interaction with the host. Fibronectin type III domain‐containing protein and mainly ankyrin repeat domains are enriched in sponge‐symbiotic community in marine sponges compared to free‐living communities (Thomas et al. 2010; Fan et al. 2012; Fiore et al. 2015; Liu et al. 2016; Sugden et al. 2022). These eukaryotic‐like domains presumably facilitate interaction with host cells or proteins, although the mechanisms for this are not well characterised. All of these traits would be expected in symbiotic bacteria and were observed to decrease in abundance in the *Flavobacterium* genus in Post samples, suggesting that some of these bacteria may be facultative symbionts that either leave the sponge or decrease their metabolism, but further work to unravel this particular sponge‐microbe association is warranted.

The sponges collected for metatranscriptome analysis were collected over 3 months in summer and early fall where the temperature, irradiance levels, turbidity, and water levels change in addition to physiological changes in the sponge. Environmental parameters of pH and temperature did not appear to vary visibly over the 3 months at the New River collection site, but conductivity did increase over time and could be a factor for gemmule formation. There was also a decrease in both bacterial cells and phytoplankton cells in the river water from August to September, which could also be a factor for gemmule formation. The differences in environmental parameters could also affect the resident microbial community in both the water and the sponge, and it is difficult or impossible to fully tease apart such impacts on our results.

In summary, the results described here suggest that the majority of the microbial community of *Radiospongilla crateriformis* does not significantly change as the sponge progresses through gemmule formation and tissue degradation; however, there is support for the presence of a few symbiotic bacterial taxa in the sponge with potential for obligate or long‐term symbiosis that could be important throughout the sponge life cycle. Future work is needed to track sponge microbiome communities over time and between species and compare the phylogeny of these taxa with other freshwater sponge taxa. Despite limitations of the experimental work here, we have generated several hypotheses for future work with this system for microbiome questions and laid a foundation for future experiments with gemmules and targeted molecular and/or culture work on specific bacterial groups.

## Author Contributions

**C.L.F.** conceived and designed the experiment and supervised the experimental work and reviewed the manuscript. **T.A.S.** provided input on the experimental design and led the experimental work and data analysis and wrote the first draft of the manuscript. **C.G.E.** provided input on the experimental design and data analysis and reviewed the manuscript.

## Funding

This work was supported by Appalachian State University and National Science Foundation (1924540).

## Ethics Statement

All coauthors have approved this manuscript for submission to Environmental Microbiology Reports. These data are not under consideration for publication elsewhere.

## Conflicts of Interest

The authors declare no conflicts of interest.

## Supporting information


**File S1:** Supplemental methods with SAMSA2 details and supplemental Figures S1 through S4.
**Figure S1:** Example images of sponge morphology and structure. Brightfield microscopy image of sample NR58 at 400× total magnification with megascleres in the centre and one gemmulosclere on the right (A). The megascleres have subtle spines and the gemmulosclere is birotule with spines at the distal ends of the shaft (A). Example of sponge NR55 prior to sample collection (B), and an example of a sponge with gemmules present, but not one from this study (C). White arrows in C point to gemmules (light coloured spheres).
**Figure S2:** Relative abundance of reads assigned to the genus *Sediminibacterium* (Phylum Bacteroidota). Taxonomy is derived from taxonomic annotation of RNA reads using the RefSeq prokaryotic database. Pre and Post labels correspond to sponges pre‐ and post‐gemmule formation in sponges.
**Figure S3:** The log2 fold change in abundance for taxa identified as differentially abundant taxa from DEseq2 analysis of RNA reads. Pre samples were used as the control, so a positive fold change indicates taxa that increased in abundance in Post samples, and a negative fold change indicates taxa that decreased in abundance in the Post samples. Taxonomy was derived from taxonomic annotation of RNA reads using the RefSeq prokaryotic database.
**Figure S4:**. Relative abundance of functional genes with 10% or greater abundance in each sample in the metatranscriptome. Function is derived from annotation of RNA reads using the RefSeq prokaryotic database. Pre and Post labels correspond to sponges pre‐ and post‐gemmule formation in sponges. Each pie chart represents a sample from Pre and Post stages.

**Table S1:** Tracking of reads through cleaning steps of the SAMSA2 pipeline.

**Table S2:** Number of reads that mapped the freshwater sponge Ephydatia muelleri and to the Refseq Prokaryotic database.

**Table S3:** Diversity index results for organism and functional analyses of RNA reads processed through the SAMSA2 pipeline. Taxonomy and function of the reads were assigned from the RefSeq prokaryotic database.

**Table S4:** Operational taxonomic units (OTU) identified by RefSeq assigned annotations to RNAseq reads as part of the SAMSA2 pipeline in order of taxonomy. These include taxa that accounted for 90% of the total abundance from SAMSA2 output and were converted to relative abundances.

**Table S5:** Organisms that were significantly different in abundance between the pre‐ and post‐gemmule sponge samples. Differential abundance was calculated using DESeq2 (Pre was used as the control group) and the table includes sample group mean abundances, log2 fold change, which indicates if the organisms increased or decreased in abundance, the error associated with the fold change (lfcSE), the statistic (stat), and adjusted *p*‐value.

**Table S6:** Table of functions identified by RefSeq assigned annotation to RNAreads as part of SAMSA2 pipeline. Functions that accounted for 90% of the total abundances from SAMSA2 output were convered to relative abundances to populate this table and results were manually compiled here with the corresponding KEGG ontology (M1‐M4).

**Table S7:** Functions that were significantly different in abundance between the pre and post gemmule sponge samples. Differential abundance was calculated using DESeq2 and the table includes sample means, log2 fold change, indicating if the functions increased or decreased in abundance, the error associated with the fold change (lfcSE), as well as the statistic (stat), and adjusted *p*‐value.

**Table S8:** Functions that were significantly different in abundance between the pre‐ and post‐gemmule sponge samples from two genera (Polynucleobacter and Flavobacterium). Differential abundance was calculated using DESeq2 and the table includes sample means, log2 fold change, indicating if the functions increased or decreased in abundance, the error associated with the fold change (lfcSE), as well as the statistic (stat), and adjusted *p*‐value.

## Data Availability

Sequencing data are available through NCBI SRA BioProject PRJNA976703 and environmental data are available at OSF DOI: https://doi.org/10.17605/OSF.IO/ZURST.
