# Supplementary material for: Low Abundance Taxa Show Diverse Microbial Symbiotic Interactions With the Freshwater Sponge, Radiospongilla crateriformis , Pre and Post Gemmulation
Source: Environ Microbiol Rep. 2026 Apr 6;18(2):e70331. doi: 10.1111/1758-2229.70331 (PMC13052501; doi:10.1111/1758-2229.70331)
Supplement: Supplementary file 1 — File S1: Supplemental methods with SAMSA2 details and supplemental Figures S1 through S4. Figure S1: Example images of sponge morphology and structure. Brightfield microscopy image of sample NR58 at 400× total magnification with megascleres in the centre and one gemmulosclere on the right (A). The megascleres have subtle spines and the gemmulosclere is birotule with spines at the distal ends of the shaft (A). Example of sponge NR55 prior to sample collection (B), and an example of a sponge with gemmules present, but not one from this study (C). White arrows in C point to gemmules (light coloured spheres). Figure S2: Relative abundance of reads assigned to the genus Sediminibacterium (Phylum Bacteroidota). Taxonomy is derived from taxonomic annotation of RNA reads using the RefSeq prokaryotic database. Pre and Post labels correspond to sponges pre‐ and post‐gemmule formation in sponges. Figure S3: The log2 fold change in abundance for taxa identified as differentially abundant taxa from DEseq2 analysis of RNA reads. Pre samples were used as the control, so a positive fold change indicates taxa that increased in abundance in Post samples, and a negative fold change indicates taxa that decreased in abundance in the Post samples. Taxonomy was derived from taxonomic annotation of RNA reads using the RefSeq prokaryotic database. Figure S4:. Relative abundance of functional genes with 10% or greater abundance in each sample in the metatranscriptome. Function is derived from annotation of RNA reads using the RefSeq prokaryotic database. Pre and Post labels correspond to sponges pre‐ and post‐gemmule formation in sponges. Each pie chart represents a sample from Pre and Post stages. [file EMI4-18-e70331-s002.docx]

**Supplemental information for: Low Abundance Taxa Show Diverse Microbial Symbiotic Interactions with the Freshwater Sponge, *Radiospongilla crateriformis*, Pre and Post Gemmulation**

**Taylor A. Strope^1,2^, Cole G. Easson^3^, Cara L. Fiore^1^**

**^1^Department of Biology, Appalachian State University, Boone, North Carolina, USA**

**^2^Current address: Department of Biochemistry and Molecular Biology, University of Kansas Medical Center, Kansas City, Kansas, USA (tstrope@kumc.edu)**

**^3^Department of Biology, Middle Tennessee State University, Murfreesboro, Tennessee, USA (cole.easson@mtsu.edu)**

**Corresponding author:**

**Cara Fiore**

**fiorec@appstate.edu**

**Metatranscriptome analysis with SAMSA2**

*Preprocessing*

RNAseq produced forward and reverse reads for each RNA (cDNA) that was sequenced, therefore the first step in preprocessing was to merge paired reads. PEAR 0.9.8 was used to merge paired reads (Zhang et al. 2014), which produced a single file of extended fragments from the two (forward and reverse) FASTQ sequencing files per sample. The command for PEAR was:

$pear_location/pear -f $forward_reads.fastq -r $reverse_reads.fastq -o $out_name

Where, -f points to the forward reads, -r points to the reverse reads, and -o designates the output file of merged reads. Next, low-quality sequences and/or adaptor contamination were removed using the program Trimmomatic 0.36 (Bolger et al. 2014). Trimmomatic was specifically designed to be used for Illumina sequences and thus includes the adapter and primer sequences for these machines. Trimmomatic was also used to remove low quality reads (below phred = 33) or trim the reads to produce an average phred score of 33. The command for Trimmomatic was as follows:

java -jar $./trimmomatic-0.36.jar SE -phred33 $infile $outfile_name SLIDINGWINDOW:4:15 MINLEN:99

Where, SE is the flag for single end mode (only one input file-the merged reads file from PEAR), -phred33- specifies the base quality encoding (FASTQ quality score), SLIDINGWINDOW- <windowSize>:<requiredQuality>, and MINLEN- specifies the minimum length for reads to be kept. For the sliding window, the ‘windowSize’ specifies the number of bases to produce an average as the program moves across the read, and ‘requiredQuality’ specifies the average quality threshold.

A ‘cleaned’ output file for each sample is produced from Trimmomatic for the final preprocessing step. Although a prior ribodepletion has already been performed, due to the naturally high rRNA counts present in microbiome samples, a digital ribodepletion is included in the SAMSA2 pipeline as the final preprocessing step. SortMeRNA 2.1 (Kopylova et al. 2012) removed any ribosomal reads left in the samples that may affect annotation of the metatranscriptome. SortMeRNA incorporates several reference databases for both bacterial and eukaryotic sequences for rRNA identification: SILVA, GreenGenes, and Ribosomal Database Project (RDP). The command for SortMeRNA was as follows:

$sortmerna_location/sortmerna --ref $sortmerna_location/rRNA_databases/silva-bac-16s-id90.fasta,$sortmerna_location/index/silva-bac-16s-db --reads $file --aligned $file.ribosomes --other $file.ribodepleted --fastx --num_alignments 0 --log -v

Where, --ref points to the FASTA reference file, --reads points to the FASTA/FASTQ reads files, --aligned points to the aligned reads filepath, --other points to the rejected reads filepath, --fastx indicates to make the output file in a FASTA/FASTQ format, --num_alignments 0 indicates all alignments reaching the E-value threshold were reported; very slow, --log created an output of overall statistics, and -v stands for verbose. Two files were produced: $file.ribosomes which contains all the rRNA sequences, and $file.ribodepleted which contain all the reads not identified as ribosomes. The ribodepleted file was used in the annotation step.

*Annotation*

SAMSA2 used DIAMOND (Buchfink et al. 2015) for the annotation process with the NCBI bacterial RefSeq database (Tatusova et al. 2014) downloaded in March of 2020. The commands for DIAMOND were as follows: to perform the annotation process and to covert the generated results file into a data table for the aggregation step, respectively.

1. $diamond_location blastx --db $diamond_database -q $file -a $file.RefSeq -t ./ -k 1 –sensitive
2. $diamond_location view --daa $file.RefSeq.daa -o $shortname -f tab

Where, in command 1 , -db specifies the reference database to use, -q indicates the query file name, -a indicates the name of results file in DIAMOND format, -t sets the temporary directory locations needed as the program runs, -k 1 indicates the number of hits above cutoff (e-value cutoff = 0.001) to return, and settings changed from “–fast” to “–sensitive” to increase accuracy. In command 2, --daa points to the output file in .daa format, -o determines the output file name, and -f indicates separator of values in final output.

*Aggregation and downstream processing*

DIAMOND produced annotated files with each sequence from the metatranscriptome that had a corresponding match with a reference base sequence occupied one line in the output file. The next step used SAMSA2-derived Python 2.7 scripts to aggregate these line-by-line files into condensed, summary tables (Westreich et al., 2018). This aggregation step created two files from the same DIAMOND output reads: one for organism annotations and the other for functional annotations into 3 column tables. Two commands were used to generate the aggregation output files:

1. python $python_programs/standardized_DIAMOND_analysis_counter.py -I $file -D $RefSeq_db -O
2. python $python_programs/standardized_DIAMOND_analysis_counter.py -I $file -D $RefSeq_db -F

Where, -I points to the infile, -D indicates the specific reference database to search against, -O (in command 3) indicates to aggregate all reads by organism, and -F (in command 4) indicates to aggregate all reads by function.

*Additional applications*

We also used the SAMSA2 scripts for annotating function by specific organisms (“DIAMOND_specific_organism_retreiver.py”). This is possible because each processed read already receives both an organism and functional annotation. The command for the specific organism retriever was:

python $python_programs/DIAMOND_specific_organism_retriever.py -I $file -SO (genus or species) -D $RefSeq_db

Where, -I points to the input file, which is the DIAMOND results file from annotation step above, -SO specifics which specific organism, either genus or species, to be isolated, and -D specifies the reference database to use. This created an output file containing all the individual transcripts that originated from the specified organism. Following this, the produced file was aggregated using the command:

python $python_programs/DIAMOND_analysis_counter.py -I $file -D $RefSeq_db -F

Where -I points to the infile, -D indicates the specific reference database to search against, and -F indicates to aggregate all reads by function. These files were saved as the final product and used for further statistical analysis or plotting using R (Westreich et al., 2018).

**References**

**Bolger AM, Lohse M, Usadel B.** **2014.** Trimmomatic: a flexible trimmer for Illumina sequence data. *Bioinformatics* **30(15)**:2114-2120.

**Buchfink B, Xie C, Huson DH.** **2015.** Fast and sensitive protein alignment using DIAMOND. *Nature Methods* **12(1)**:59-60.

**Kopylova E, Noe L, Touzet H.** **2012.** SortMeRNA: fast and accurate filtering of ribosomal RNAs in metatranscriptomic data. *Bioinformatics* **28(24)**:3211-3217.

**Tatusova T, Ciufo S, Fedorov B, O’Neill K, Tolstoy I.** **2014.** RefSeq microbial genomes database: new representation and annotation strategy. *Nucleic Acids Research* **42(Database issue)**:D553-559.

**Westreich ST, Treiber ML, Mills DA, Korf I, Lemay DG. 2018.** SAMSA2: a standalone metatranscriptome analysis pipeline. *BMC Bioinformatics* **19**:175 DOI 10.1186/s12859-018-2189-z.

**Zhang J, Kobert K, Flouri T, Stamatakis A.** **2014.** PEAR: a fast and accurate Illumina paired-end reAd mergeR. *Bioinformatics (Oxford, England)* **30(5)**:614-620.

Supplemental Figures:


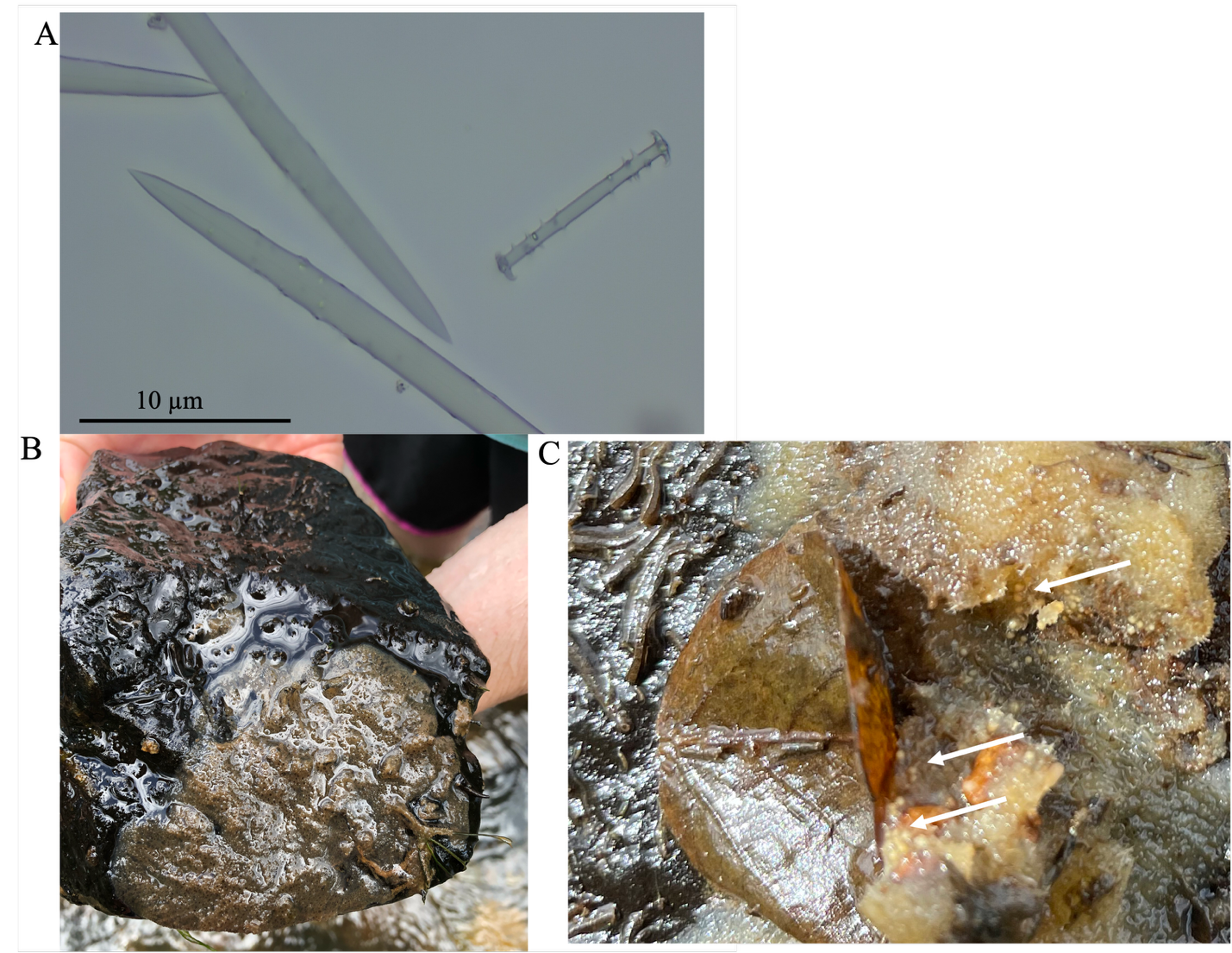


**Figure S1.** Example images of sponge morphology and structure. Brightfield microscopy image of sample NR58 at 400x total magnification with megascleres in the center and one gemmulosclere on the right (A). The megascleres have subtle spines and the gemmulosclere is birotule with spines at the distal ends of the shaft (A). Example of sponge NR55 prior to sample collection (B), and an example of a sponge with gemmules present, but not one from this study (C). White arrows in C point to gemmules (light colored spheres).

**
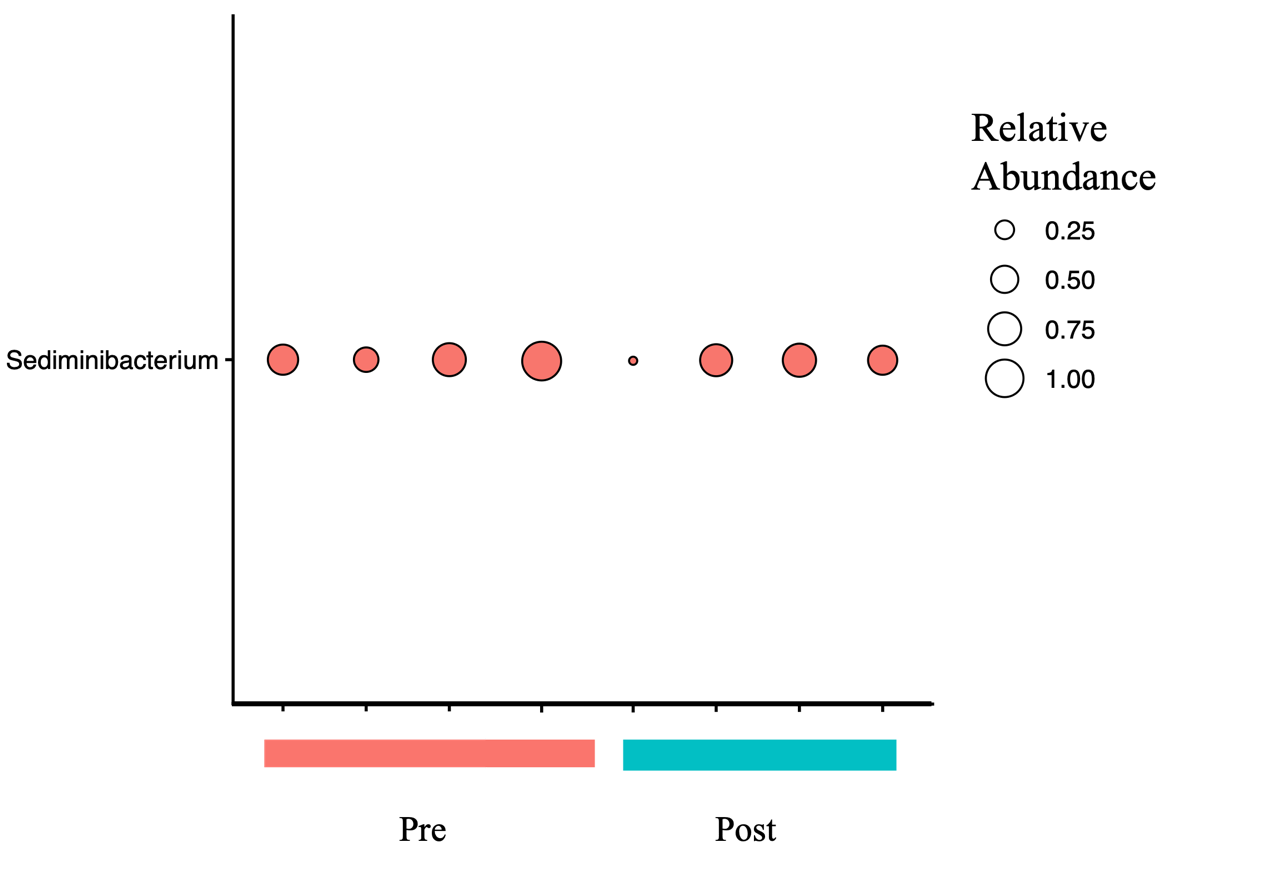
**

*Sediminibacterium*

**Figure S2.** Relative abundance of reads assigned to the genus *Sediminibacterium* (Phylum Bacteroidota). Taxonomy is derived from taxonomic annotation of RNA reads using the RefSeq prokaryotic database. Pre and Post labels correspond to sponges pre- and post-gemmule formation in sponges.

**
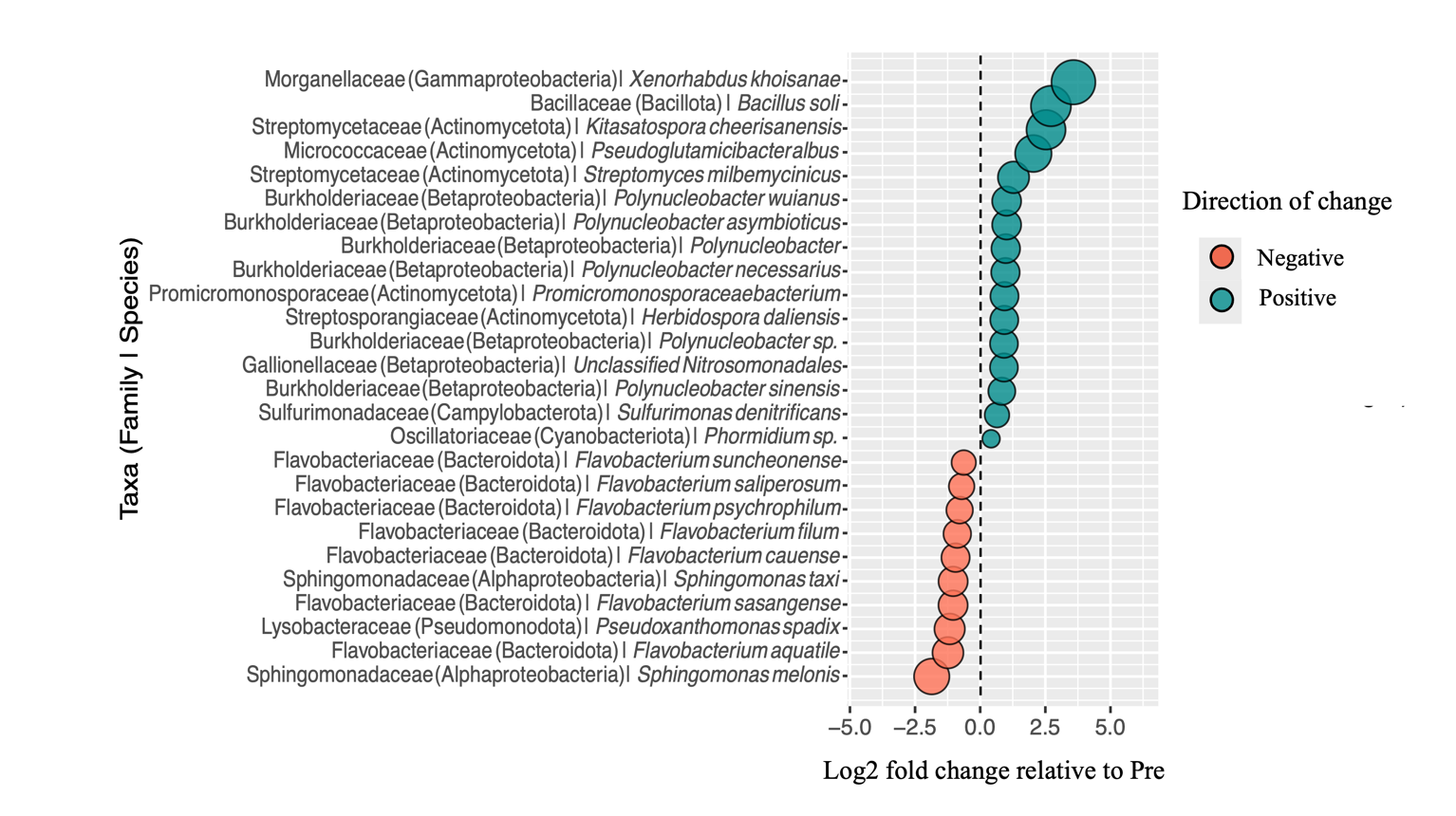
**

**Figure S3.** The log2 fold change in abundance for taxa identified as differentially abundant taxa from DEseq2 analysis of RNA reads. Pre samples were used as the control, so a positive fold change indicates taxa that increased in abundance in Post samples, and a negative fold change indicates taxa that decreased in abundance in the Post samples. Taxonomy was derived from taxonomic annotation of RNA reads using the RefSeq prokaryotic database.


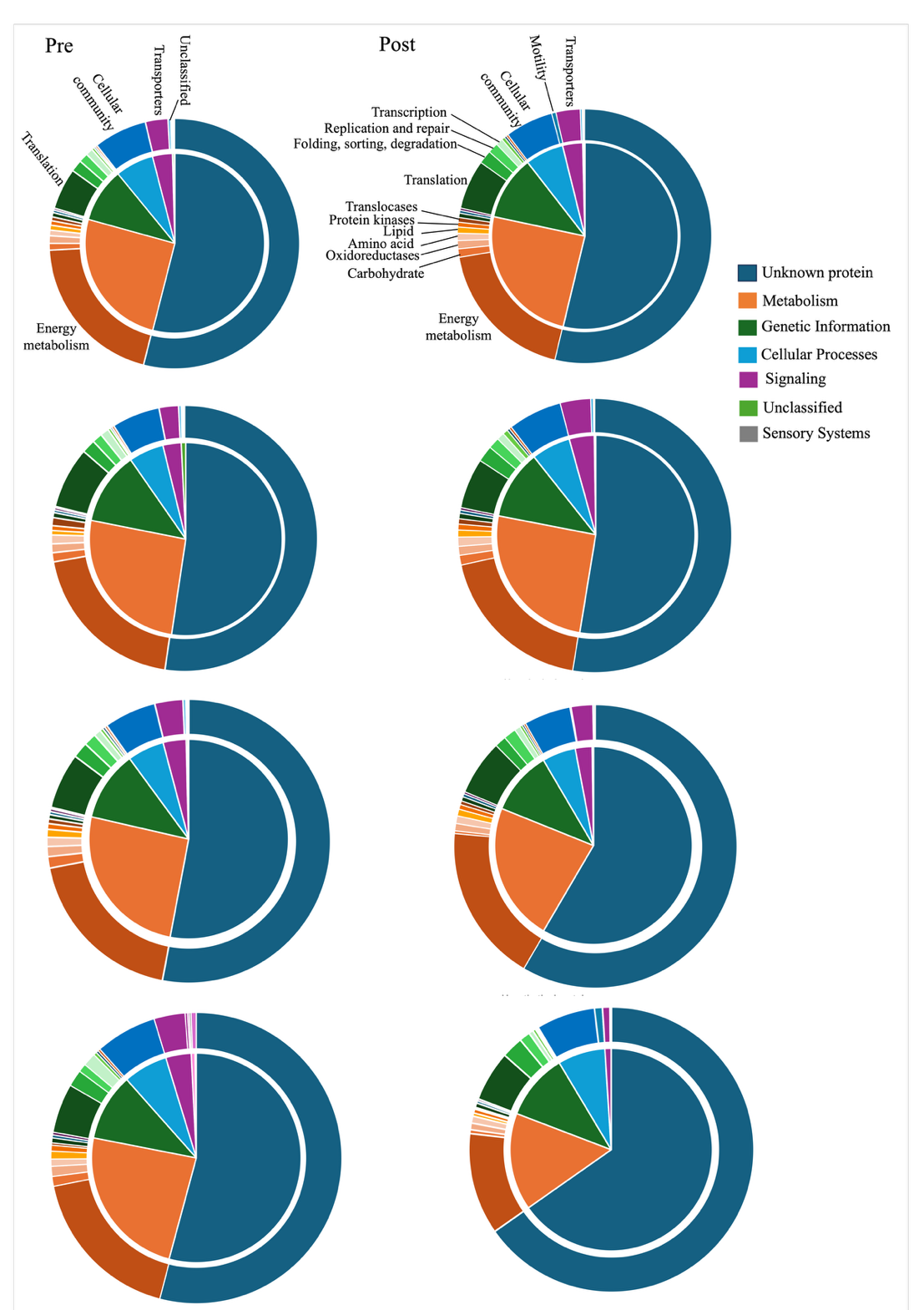


**Figure S4.** Relative abundance of functional genes with 10% or greater abundance in each sample in the metatranscriptome. Function is derived from annotation of RNA reads using the RefSeq prokaryotic database. Pre and Post labels correspond to sponges pre- and post-gemmule formation in sponges. Each pie chart represents a sample from Pre and Post stages.
